# A compartmental model that predicts the effect of social distancing and vaccination on controlling COVID-19

**DOI:** 10.1038/s41598-021-86873-0

**Published:** 2021-04-14

**Authors:** Mohammadali Dashtbali, Mehdi Mirzaie

**Affiliations:** Department of Applied Mathematics, Faculty of Mathematical Sciences, Tarbiat Modares University, Jalal Al-Ahmad High Way, Tehran, Iran

**Keywords:** Mathematics and computing, Infectious diseases, Viral infection

## Abstract

The understanding of the interaction between disease dynamics and human behavior is an important and essential point to control infectious. Disease outbreak can be influenced by social distancing and vaccination. In this study, we introduce two compartmental models to derive the epidemic curve and analyze the individual’s behavior in spreading and controlling the COVID-19 epidemic. The first model includes Susceptible, Exposed, Infectious, Hospitalized, Recovered and Death compartments and in the second model, we added a new compartment namely, semi-susceptible individuals that are assumed to be more immune than the susceptible. A comparison of the two models shows that the second model provides a better fit to the daily infected cases from Egypt, Belgium, Japan, Nigeria, Italy, and Germany released by WHO. Finally, we added a vaccinated term to the model to predict how vaccination could control the epidemic. The model was applied on the record data from WHO.

## Introduction

During history, the human encountered with severe pandemics such as the Spanish flu in 1917, the Honk Kong flu (H3N2) in 1968, and the swine flu (H1N1) in 2009 affecting people on a worldwide scale^1^. Recently, in December, 2019, the first case of the Corona Virus Disease (COVID-19) was identified in Wuhan, China and since then has spread to almost all countries and regions around the world^2^. The COVID-19 pandemic, as the most crucial global health disaster of the century infected millions of people and leads to thousands of deaths over the world. The disease caused by a new betacoronavirus, severe acute respiratory syndrome coronavirus 2 (SARS-COV-2) related to the middle east respiratory syndrome virus (MERS-CoV) and the severe acute respiratory syndrome virus (SARS-CoV). This virus spreads mainly between individuals through close contacts by respiratory droplets produced when an infected person coughs, sneezes or even talks. The disease is highly transmittable with specific characteristics including the incubation period and high infectivity during incubation. The incubation period is 2–14 days and during this time when no symptoms are present on the patients^3, 4^.

Several mathematical models have been introduced to analyze the dynamics of infectious diseases to predict the number of infected, recovered, and removed in the future. In 1927, Kermack and McKendrick^5^ presented one of the first models to forecast the progress of the epidemic. In this model, a population is divided into distinct compartments and assumed that individuals in every compartment, have the same characteristics. The model is called SIR, consists of three compartments that divide a given population into susceptible (S), infected (I), and recovered (R) individuals and a system of three differential equations describe the dynamics of disease during time and the individuals’ transition in a population between different compartments. Ever since, several simple and complex compartmental models have been introduced to analyze different types of infectious disease and predict the duration of an epidemic, the prevalence and finding how control interventions such as social distancing and immunization, as well as vaccination could reduce the epidemic size. These can be taken as useful measures to evaluate the effectiveness of control policies and actions against the spreading of infectious diseases. Recently, several dynamical models have been presented to describe the dynamics of the evolution of COVID-19 and trying to predict the duration of epidemics and the number of infected individuals in different countries^6–13^. Note that COVID-19 as a disease caused by a new virus needs a model taking into consideration its known specific characteristics.

Since the disease spreads through the close interaction, the social distancing and reducing contact in population was suggested to control the epidemic. The presented model also should consider the effect of social distancing on the dynamics of the disease. The concept of social distancing as a nonpharmaceutical action to reduce and control the spread of an epidemic was studied by Reluga^14^. According to optimal control and differential game approaches, the impact of social distancing in the spread of infectious diseases on the network was studied in 2020^15^.

Vaccination policy is related to the population health adopted by the government. Eradicating the disease or the immunity of the population is the aim of vaccination. Individuals’ deciding about vaccination is involved considering the risk of morbidity from vaccination and the probability of getting infected^16–19^. On the other hand, each person’s decisions in vaccination are influenced by the decisions of others in social distancing^20, 21^. In this study, two models have been studied. The first model consists of seven compartments including the Susceptible (S), Exposed (E), Infected (I), Hospitalized (H), Recovered (R), and Death (D) individuals, called SEIHRD. After getting infected, an individual enters the hospitalized compartment and at the end of this state either recovers or dies. The model also considers social distancing and we assume that number of dead people among the total number of infected individuals could affect the behavior of people in social distancing. Some researches indicate that some people are less likely to become infected. In the second model, we added a compartment, Semi-susceptible (M) and assume that these individuals are half as susceptible to COVID-19. The new model is called SMEIHRDV model. The results of the two models were compared using WHO data from Japan, Italy, Belgium, Germany, Nigeria and Egypt. The second model was fitted better than the first one. Finally, the SMEIHRDV model was simulated for the different coverages of vaccination to predict how vaccination can control the epidemic.

The main purpose of this study is to develop a compartmental model to fit the released data by WHO and could predict the behavior of epidemic. This model can be used to investigate how vaccination coverage will control the epidemic. Social distancing is one of the best strategies limiting contact rate and reducing transmission rates. A differential game theory model also introduced to investigate the role of individual’s investment in social distancing for controlling the epidemic. The organization of this paper is as follows: in “SEIHRDV model” we present the SEIHRDV model. In “SMEIHRDV model”, the SEIHRDV model is expanded to the SMEIHRDV model. The game theory of social distancing is considered in “The game theory of social distancing”. Next, results and discussion are presented in “Results and discussion”.

## SEIHRDV model

The most commonly implemented dynamical model in infectious disease is the SIR model, consists of three compartments including susceptible, infected and recovered individuals represented by S, I and R, respectively. In order to represent the number of susceptible, infected and recovered individuals with respect to time, these variables are considered as function of t (time): ${\mathrm{S}}({\mathrm{t}}), {\mathrm{I}}({\mathrm{t}})$ and $\mathrm{R}(\mathrm{t}).$ The dynamics of SIR model is as follows:

1 $$\begin{aligned} \frac{{\mathrm{dS}(\mathrm{t})}}{{\mathrm{dt}}}=&\, {} - \upbeta \frac{\mathrm{S}(\mathrm{t})}{\mathrm{NI}}(\mathrm{t}),\nonumber \\ \frac{{{\mathrm{dI}(\mathrm{t})}}}{{{\mathrm{dt}}}}=&\, {} \upbeta \frac{{\mathrm{S}(\mathrm{t})}}{\mathrm{NI}}(\mathrm{t}) - \upgamma \mathrm{I}(\mathrm{t}),\nonumber \\ \frac{{\mathrm{dR}(\mathrm{t})}}{{\mathrm{dt}}}=&\, {} \upgamma \mathrm{I}(\mathrm{t}). \end{aligned}$$

**Figure 1:**
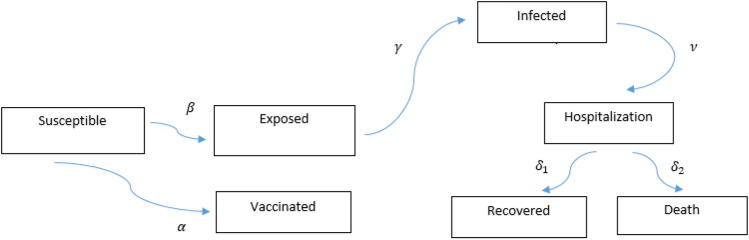
SEIHRDV model. The diagram for SEIHRDV model that S, E, I, H, R, D and V are the number of susceptible individuals, exposed individuals (persons in the incubation period after being infected by the disease pathogen), infected individuals, hospitalized individuals, recovered individuals, death individuals and vaccinated individuals, respectively.

Letting total population size denoted by $\mathrm{N}$, is constant and $\mathrm{N} =\mathrm{S}(\mathrm{t}) + \mathrm{I}(\mathrm{t})+ \mathrm{R}(\mathrm{t})$. Several compartmental models have been extended from the SIR model. According to the reported characteristics of the COVID-19 disease, we develop a mathematical model consists of seven compartments:

1. Susceptible individuals denoted by S who are not infected by the disease pathogen,
2. Exposed individuals denoted by E. The persons in the incubation period after being infected by the virus. They have no visible clinical signs but infectious.
3. Infected individuals denoted by I. After the incubation period, it is the first compartment of the infectious period, where the person has finished the incubation period, may infect other people and starts developing clinical signs. After this period, people needed to be hospitalized (in a hospital or at home), therefore the next state is:
4. Hospitalized individuals denoted by H. After the hospitalization period, people can be, either recovered or removed. Therefore, we have two other compartments:
5. Recovered individuals denoted by R , and
6. Death individuals denoted by D. In order to predict the effect of vaccination, after releasing the vaccine, we considered the vaccinated compartment. So:
7. Vaccinated individuals denoted by V. The suggested dynamics of this model (called SEIHRDV) is as follows:

2 $$\begin{aligned} \frac{{\mathrm{dS}(\mathrm{t})}}{{\mathrm{dt}}}=& \,{} -(1 - \upalpha )\upbeta \frac{\mathrm{S}(\mathrm{t})}{\mathrm{NI}}(\mathrm{t}) - \upalpha \mathrm{S}(\mathrm{t}),\nonumber \\ \frac{{\mathrm{dE}(\mathrm{t})}}{{\mathrm{dt}}}=&\, {} (1 - \upalpha )\upbeta \frac{\mathrm{S}(\mathrm{t})}{\mathrm{NI}}(\mathrm{t}) - \upgamma \mathrm{E}(\mathrm{t}),\nonumber \\ \frac{{\mathrm{dI}(\mathrm{t})}}{{\mathrm{dt}}}=& \,{} \upgamma \mathrm{E}(\mathrm{t}) - \upnu \mathrm{I}(\mathrm{t}),\nonumber \\ \frac{{\mathrm{dH}(\mathrm{t})}}{{\mathrm{dt}}}=&\, {} \upnu \mathrm{I}(\mathrm{t}) - {{\updelta } _{1H}}(\mathrm{t}) - {{\updelta } _{2H}}(\mathrm{t}),\nonumber \\ \frac{{\mathrm{dR}(\mathrm{t})}}{{\mathrm{dt}}}=& \,{} {{\updelta } _{1H}}(\mathrm{t}),\nonumber \\ \frac{{\mathrm{dD}(\mathrm{t})}}{{\mathrm{dt}}}=&\, {} {{\updelta } _{2H}}(\mathrm{t}),\nonumber \\ \frac{{\mathrm{dV}(\mathrm{t})}}{{\mathrm{dt}}}=&\, {} \upalpha \mathrm{S}(\mathrm{t}), \end{aligned}$$

where,

- $\upbeta$ denotes the infection rate,
- ${\upgamma ^{ - 1}}$ is the average latent time,
- ${{\updelta } _{1}}$ is the cure rate,
- ${{\updelta } _{2}}$ is the mortality rate,
- $\upalpha$ is vaccine coverage.
- $\upnu$ is the hospitalized rate

and $\mathrm{N}=\mathrm{S}(\mathrm{t})+\mathrm{E}(\mathrm{t})+\mathrm{I}(\mathrm{t})+\mathrm{H}(\mathrm{t})+\mathrm{R}(\mathrm{t})+\mathrm{D}(\mathrm{t})+\mathrm{V}(\mathrm{t})$ where $\mathrm{N}$ is the number of people in the population. The diagram for this model is depicted in Fig. 1.

### Social distancing

Social distancing is a non-pharmaceutical strategy to reduce and control the spread of an epidemic^14^. It could decrease the transmission rate between susceptible and infected individuals and affects the dynamics of an epidemic. In this study, we consider behavior of individuals by simple function of the available information. In fact we assume that susceptible individuals change their contact rate based on the number of confirmed death per day in a country. Also, in order to investigate the effectiveness of social distancing during time, we use function $\upsigma (\mathrm{t})$ as it was discussed by Reluga^14^. Therefore, the system of Eq. (2) is modified as follows:

3 $$\begin{aligned} \frac{{\mathrm{dS}(\mathrm{t})}}{{\mathrm{dt}}}=& \,{} - \upsigma (\mathrm{t})(1 - \upalpha )\upbeta \frac{\mathrm{S}(\mathrm{t})}{\mathrm{NI}}(\mathrm{t}) - \upalpha \mathrm{S}(\mathrm{t}),\nonumber \\ \frac{{\mathrm{dE}(\mathrm{t})}}{{\mathrm{dt}}}=&\, {} \upsigma (\mathrm{t})(1 - \upalpha )\upbeta \frac{\mathrm{S}(\mathrm{t})}{\mathrm{NI}}(\mathrm{t}) - \upgamma \mathrm{E}(\mathrm{t}),\nonumber \\ \frac{{\mathrm{dI}(\mathrm{t})}}{{\mathrm{dt}}}=& \,{} \upgamma \mathrm{E}(\mathrm{t}) - \upnu \mathrm{I}(\mathrm{t}),\nonumber \\ \frac{{\mathrm{dH}(\mathrm{t})}}{{\mathrm{dt}}}=&\, {} \upnu \mathrm{I}(\mathrm{t}) - {{\updelta }_{1H}}(\mathrm{t}) - {{\updelta } _{2H}}(\mathrm{t}),\nonumber \\ \frac{{\mathrm{dR}(\mathrm{t})}}{{\mathrm{dt}}}=& \,{} {{\updelta } _{1H}}(\mathrm{t}),\nonumber \\ \frac{{\mathrm{dD}(\mathrm{t})}}{{\mathrm{dt}}}=&\, {} {{\updelta } _{2H}}(\mathrm{t}),\nonumber \\ \frac{{\mathrm{dV}(\mathrm{t})}}{{\mathrm{dt}}}=&\, {} \upalpha \mathrm{S}(\mathrm{t}), \end{aligned}$$

where $\upsigma (\mathrm{t})$ estimated using the number of confirmed death per day in a country and is $\upsigma (\mathrm{t})=\frac{1}{\mathrm{p}(1+\mathrm{c}(\mathrm{t}))}$ where $\mathrm{c}(\mathrm{t})$ is, the ratio of the number of daily died COVID-19 patients at time $\mathrm{t}$ to the number of daily diagnosed COVID-19 patients at time $\mathrm{t}$. In order to consider individual heterogeneities such as differences in social activity, mixing patterns including household, spatial aspects, and travels, we used a parameter, namely p, in the model which is calibrated to fit the model to data. The selected values of ${\mathrm{p} ^{ - 1}}$ for different countries are as follows: Egypt: 0.47, Germany: 0.50, Belgium: 0.54, Japan: 0.55, Italy: 0.56, Nigeria: 0.57.

## SMEIHRDV model

**Figure 2:**
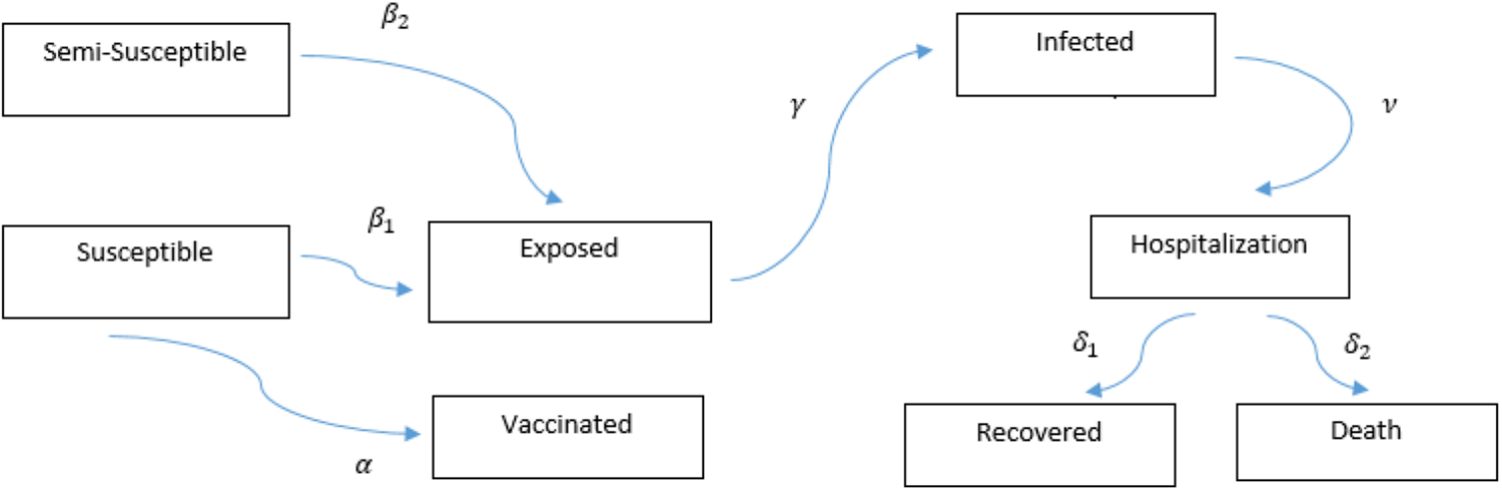
SMEIHRDV model. SMEIHRDV model that S, M, E, I, H, R, D and V are the number of susceptible, exposed, infected, semi-susceptible, hospitalized, recovered, death, and vaccinated individuals, respectively.

Elderly and sick people are most susceptible to severe forms of COVID-19. There have been studies to discover why some individuals are more susceptible than the others^22^. For example, some studies show that people carrying certain variants of the ACE2 gene would be protected against the COVID-19 infection^23, 24^. Therefore we divided susceptible individuals into two compartments, the susceptible compartment denoted by S and the semi-susceptible denoted by M. In compartment S, people are more susceptible than M. The new model is called the SMEIHRDV model. A diagram summarizing the main structure of our model is presented in Fig. 2. ${{\upbeta } _{1}}$ is the transmission rate between susceptible and infected individuals and ${{\upbeta } _{2}}$ is the transmission rate between semi-susceptible and infected individuals. The remaining parameters are the same as the SEIHRDV model. The dynamics of this model is as follows:

4 $$\begin{aligned} \frac{{{\text{dS}}({\text{t}})}}{{{\text{dt}}}} =&\, - (1 - \alpha )(\sigma ({\text{t}}))\beta _{1} \frac{{{\text{S}}({\text{t}})}}{{{\text{NI}}}}({\text{t}}) - \alpha {\text{S}}({\text{t}}), \\ \frac{{{\text{dM}}({\text{t}})}}{{{\text{dt}}}} =&\, - \beta _{2} (\sigma ({\text{t}}))\frac{{{\text{M}}({\text{t}})}}{{{\text{NI}}}}({\text{t}}), \\ \frac{{{\text{dE}}({\text{t}})}}{{{\text{dt}}}} =&\, (1 - \alpha )\beta _{1} \frac{{{\text{S}}({\text{t}})}}{{{\text{NI}}}}({\text{t}}) + \beta _{2} \frac{{{\text{M}}({\text{t}})}}{{{\text{NI}}}}({\text{t}}) - \gamma {\text{E}}({\text{t}}), \\ \frac{{{\text{dI}}({\text{t}})}}{{{\text{dt}}}} =& \,\gamma {\text{E}}({\text{t}}) - \nu {\text{I}}({\text{t}}), \\ \frac{{{\text{dH}}({\text{t}})}}{{{\text{dt}}}} =& \,\nu {\text{I}}({\text{t}}) - \delta _{{1H}} ({\text{t}}) - \delta _{{2H}} ({\text{t}}), \\ \frac{{{\text{dR}}({\text{t}})}}{{{\text{dt}}}} =& \,\delta _{{1H}} ({\text{t}}), \\ \frac{{{\text{dD}}({\text{t}})}}{{{\text{dt}}}} =& \,\delta _{{2H}} ({\text{t}}), \\ \frac{{{\text{dV}}({\text{t}})}}{{{\text{dt}}}} =& \,\alpha {\text{S}}({\text{t}}). \\ \end{aligned}$$

## The game theory of social distancing

Social distancing as a strategy limiting contact rates and reducing transmission rate has costs in terms of money, time and convenience. Reluga^14^ introduced a population game where the payoff of individuals is characterized by the individual’s behavioral strategy and the average behavioral strategy adopted by the population. Let $\overline{\text{c}}_{\text{S}}$ the aggregate daily investment adopted by the susceptible population and ${\mathrm{c}}_{\text{S}}$ the specific susceptible individual’s investment. The impact of social distancing is defined by a function $\upsigma ({\mathrm{c}}_{\text{S}})$ which is the relative risk of disease at daily investment $\mathrm{c}_{\mathrm{S}}$ in social distancing. Here, we consider a differential game for a susceptible individual choosing his or her best investment strategy in social distancing relative to the aggregate investment strategy of susceptible population as whole and a differential game for a semi-susceptible individual choosing his or her best investment strategy in social distancing relative to the aggregate investment strategy of semi-susceptible population as whole. The modified version of the equations with social distancing according to differential game approach is as follows:

5 $$\begin{aligned} \frac{{\mathrm{dS}(\mathrm{t})}}{{\mathrm{dt}}}=&\, {} - (1 - \upalpha )\upsigma ({{{\overline{\mathrm{c}}}}_1}){{\upbeta } _{1}}\frac{\mathrm{S}(\mathrm{t})}{\mathrm{NI}}(\mathrm{t}) - \upalpha \mathrm{S}(\mathrm{t}),\nonumber \\ \frac{{\mathrm{dM}(\mathrm{t})}}{{\mathrm{dt}}}=&\, {} - \upsigma ({{{\overline{\mathrm{c}}}}_2}){{\upbeta } _{2}}\frac{\mathrm{M}(\mathrm{t})}{\mathrm{NI}}(\mathrm{t}),\nonumber \\ \frac{{\mathrm{dE}(\mathrm{t})}}{{\mathrm{dt}}}=&\, {} (1 - \upalpha )\upsigma ({{{\overline{\mathrm{c}}}}_1}){{\upbeta } _{1}}\frac{\mathrm{S}(\mathrm{t})}{\mathrm{NI}}(\mathrm{t}) + \upsigma ({{{\overline{\mathrm{c}}}}_2}){{\upbeta } _{2}}\frac{\mathrm{M}(\mathrm{t})}{\mathrm{NI}}(\mathrm{t}) - \upgamma \mathrm{E}(\mathrm{t}),\nonumber \\ \frac{{\mathrm{dI}(\mathrm{t})}}{{\mathrm{dt}}}=&\, {} \upgamma \mathrm{E}(\mathrm{t}) - \upnu \mathrm{I}(\mathrm{t}),\nonumber \\ \frac{{\mathrm{dH}(\mathrm{t})}}{{\mathrm{dt}}}=& {} \upnu \mathrm{I}(\mathrm{t}) - {{\updelta } _{1H}}(\mathrm{t}) - {{\updelta } _{2H}}(\mathrm{t}),\nonumber \\ \frac{{\mathrm{dR}(\mathrm{t})}}{{\mathrm{dt}}}=&\, {} {{\updelta } _{1H}}(\mathrm{t}),\nonumber \\ \frac{{\mathrm{dD}(\mathrm{t})}}{{\mathrm{dt}}}=& \,{} {{\updelta } _{2H}}(\mathrm{t}),\nonumber \\ \frac{{\mathrm{dV}(\mathrm{t})}}{{\mathrm{dt}}}=&\, {} \upalpha \mathrm{S}(\mathrm{t}), \end{aligned}$$

where

1. ${\text{c}}_{1} ({\text{t}}) =\frac{{{\text{c}}_{{\text{S}}} ({\text{t}})}}{{{\text{c}}_{{\text{I}}} }}$ and ${\text{c}}_{{\text{S}}} ({\text{t}})$ the specific susceptible individual’s investment.
2. ${\text{c}}_{2} ({\text{t}}) =\frac{{{\text{c}}_{{\text{M}}} ({\text{t}})}}{{{\text{c}}_{{\text{I}}} }}$ and ${\text{c}}_{{\text{M}}} ({\text{t}})$ the specific semi-susceptible individual’s investment.
3. $\overline{{\text{c}}} _{1} ({\text{t}}) =\frac{{\overline{{\text{c}}} _{{\text{S}}} ({\text{t}})}}{{{\text{c}}_{{\text{I}}} }}$ and $\overline{{\text{c}}} _{{\text{S}}} ({\text{t}})$ the aggregate investment in social distancing by the susceptible people.
4. $\overline{{\text{c}}} _{2} ({\text{t}}) =\frac{{\overline{{\text{c}}} _{{\text{M}}} ({\text{t}})}}{{{\text{c}}_{{\text{I}}} }}$ and $\overline{{\text{c}}} _{{\text{M}}} ({\text{t}})$ the aggregate investment in social distancing by semi-susceptible individuals.
5. ${\text{c}}_{{\text{I}}}$ the cost of infection.
6. $\upsigma$ is the relative risk and $\upsigma (\overline{{\text{c}}} _{1} ({\text{t}})) = \frac{1}{{{\text{p}}(1 + \overline{{\text{c}}} _{1} ({\text{t}}))}} ,~\upsigma (\overline{{\text{c}}} _{2} ({\text{t}})) = \frac{1}{{{\text{p}}(1 + \overline{{\text{c}}} _{2} ({\text{t}}))}} .$

It is assumed that $\mathrm{N}=\mathrm{S}(\mathrm{t})+\mathrm{M}(\mathrm{t})+\mathrm{E}(\mathrm{t})+\mathrm{I}(\mathrm{t})+\mathrm{H}(\mathrm{t})+\mathrm{R}(\mathrm{t})+\mathrm{D}(\mathrm{t})+\mathrm{V}(\mathrm{t})$ where $\mathrm{N}$ is the number of people in the population and the parameters are described in Table 1. Note that if the investment in social distancing exceeds the cost of infection, then social distancing is not economical, and so $0 \le {\text{c}}_{{\text{1}}} ({\text{t}}) \le 1$ and $0 \le {\mathrm{c}_2}(\mathrm{t}) \le 1.$ The dimensionless version of the equations is obtained as follows (let ${{\upbeta } _{1}}=\upbeta ,~{{\upbeta } _{2}} ={\mathrm{b}_1}{{\upbeta } _{1}},~{{\updelta } _{1}}=\updelta ,~{{\updelta } _{2}} ={\mathrm{b}_2}{\updelta _1},~0< {\mathrm{b}_1},{\mathrm{b}_2} < 1$):

6 $$\begin{aligned} \frac{{\mathrm{dS}'(\mathrm{t})}}{{\mathrm{dt}}}= &\, {} - (1 - \upalpha )\upsigma ({{{\overline{\mathrm{c}}}}_1})\mathrm{S}'(\mathrm{t})\mathrm{I}'(\mathrm{t}) - \upalpha \mathrm{S}'(\mathrm{t}),\nonumber \\ \frac{{\mathrm{dM}'(\mathrm{t})}}{{\mathrm{dt}}}= &\, {} - \upsigma ({{{\overline{\mathrm{c}}}}_2})\mathrm{M}'(\mathrm{t})\mathrm{I}'(\mathrm{t}),\nonumber \\ \frac{{\mathrm{dE}'(\mathrm{t})}}{{\mathrm{dt}}}= &\, {} (1 - \upalpha )\upsigma ({{{\overline{\mathrm{c}}}}_1})\mathrm{S}'(\mathrm{t})\mathrm{I}'(\mathrm{t}) + \upsigma ({{{\overline{\mathrm{c}}}}_2})\mathrm{M}'(\mathrm{t})\mathrm{I}'(\mathrm{t}) - \mathrm{E}'(\mathrm{t}),\nonumber \\ \frac{{\mathrm{dI}'(\mathrm{t})}}{{\mathrm{dt}}}= & \,{} \mathrm{E}'(\mathrm{t}) - \mathrm{I}'(\mathrm{t}),\nonumber \\ \frac{{\mathrm{dH}'(\mathrm{t})}}{{\mathrm{dt}}}= &\, {} \mathrm{I}'(\mathrm{t}) - \mathrm{H}'(\mathrm{t}),\nonumber \\ \frac{{\mathrm{dR}'(\mathrm{t})}}{{\mathrm{dt}}}= &\, {} \mathrm{H}'(\mathrm{t})\nonumber \\ \frac{{\mathrm{dD}'(\mathrm{t})}}{{\mathrm{dt}}}= &\, {} \mathrm{H}'(\mathrm{t}),\nonumber \\ \frac{{\mathrm{dV}'(\mathrm{t})}}{{\mathrm{dt}}}= &\, {} \upalpha \mathrm{S}'(\mathrm{t}),\nonumber \\ \mathrm{S}'= &\, {} \frac{\upbeta }{\upgamma }\frac{\mathrm{S}}{\mathrm{N}},~\mathrm{M}'= \frac{\upbeta }{\upgamma }\frac{\mathrm{M}}{\mathrm{N}},~\mathrm{E}'= \frac{\upbeta }{\upgamma }\mathrm{E},~\mathrm{I}'= \frac{\upbeta }{\upgamma }\mathrm{I},~\mathrm{I}'= \frac{\upbeta }{\upnu }\mathrm{H}, \nonumber \\ \mathrm{R}'= &\, {} \frac{\upbeta }{\updelta }\mathrm{R},~\mathrm{D}'= \frac{\upbeta }{\updelta }\mathrm{D},~\mathrm{V}'= \frac{\upbeta }{\upgamma }\mathrm{V},~ \widehat{{\text{t}}} = \upgamma \updelta \mathrm{t} \end{aligned}$$

**Table 1:** Description of parameters.

Parameters	Description
$\mathrm{N}$	Population (million)
${\upgamma ^{ - 1}}$	Latent time (day)
${\upnu ^{ - 1}}$	Hospitalization time (day)
${\mathrm{S}_0}$	Initial susceptible cases
${\mathrm{M}_0}$	Initial semi-susceptible cases
${\mathrm{E}_0}$	Initial exposed cases
${\mathrm{I}_0}$	Initial infection cases
${\mathrm{H}_0}$	Initial hospitalized cases
${\mathrm{R}_0}$	Initial recovered cases
${\mathrm{D}_0}$	Initial death cases
${\mathrm{V}_0}$	Initial vaccinated cases

After this, the hat-notation will be dropped and the dimensionless parameters will be used. The expected present values are calculated (the expected present value is the average value one expects after accounting for the probabilities of all future events, and discounting future costs relative to immediate costs) because of the exact time spent and the precise payoff are not predicted and so we use the Markov process. For each state, the expected present values denoted by *V* and justify in the following equation^14^:

7 $$- \mathop {\text{V}}\limits^{.} = ({\text{Q}}^{{\text{T}}} - {\text{h}\mathbb{I}}){\text{V}} + {\text{v}}$$

where $\mathrm{h}$ is the discount rate. We will take $\mathrm{h}=0$ as discassed by Reluga^25^. Also, $\mathrm{v}(\mathrm{t};{\mathrm{c}_1}(\mathrm{t}),{\mathrm{c}_2}(\mathrm{t})) = ( - {\mathrm{c}_1}(\mathrm{t}), - {\mathrm{c}_2}(\mathrm{t}),-1+\frac{({\mathrm{c}_1}(\mathrm{t})+{\mathrm{c}_2}(\mathrm{t}))}{2},-1+\frac{({\mathrm{c}_1}(\mathrm{t})+{\mathrm{c}_2}(\mathrm{t}))}{2},0,0,0,0)$ and

$$\begin{aligned} \mathrm{Q}(\mathrm{t};{\mathrm{c}_1}(\mathrm{t}),{\mathrm{c}_2}(\mathrm{t})) = \begin{pmatrix} - \upsigma ({\mathrm{c}_1}(\mathrm{t}))(1-\upalpha )\mathrm{I}'-\upalpha &{}0&{}0&{}0&{}0&{}0&{}0&{}0\\ 0&{}- \upsigma ({\mathrm{c}_2}(\mathrm{t}))\mathrm{I}'&{}0&{}0&{}0&{}0&{}0&{}0\\ \upsigma ({\mathrm{c}_1}(\mathrm{t}))(1-\upalpha )\mathrm{I}'&{} \upsigma ({\mathrm{c}_2}(\mathrm{t}))\mathrm{I}'&{}-1&{}0&{}0&{}0&{}0&{}0\\ 0&{}0&{}1&{}-1&{}0&{}0&{}0&{}0\\ 0&{}0&{}0&{}1&{}-1&{}0&{}0&{}0\\ 0&{}0&{}0&{}0&{}1&{}0&{}0&{}0\\ 0&{}0&{}0&{}0&{}1&{}0&{}0&{}0\\ \upalpha &{}0&{}0&{}0&{}0&{}0&{}0&{}0\\ \end{pmatrix} \end{aligned}$$

Therefore, we have:

8 $$\begin{aligned} \frac{{ - \mathrm{d}{\mathrm{V}_{\mathrm{S}'}}(\mathrm{t})}}{{\mathrm{dt}}}= &\, {} - ((1-\upalpha )\upsigma ({\mathrm{c}_1}(\mathrm{t})){\mathrm{I}'}(\mathrm{t})+\upalpha ){\mathrm{V}_{\mathrm{S}'}}(\mathrm{t}) + (1-\upalpha )\upsigma ({\mathrm{c}_1}(\mathrm{t}))\mathrm{I}'(\mathrm{t}){\mathrm{V}_{\mathrm{E}'}}(\mathrm{t})-{\mathrm{c}_1}(\mathrm{t})\nonumber \\ \frac{{ - \mathrm{d}{\mathrm{V}_{\mathrm{M}'}}(\mathrm{t})}}{{\mathrm{dt}}}= &\, {} - \upsigma ({\mathrm{c}_2}(\mathrm{t}))\mathrm{I}'(\mathrm{t}){\mathrm{V}_{\mathrm{M}'}}(\mathrm{t}) + \upsigma ({\mathrm{c}_2}(\mathrm{t}))\mathrm{I}'(\mathrm{t})\mathrm{V}_{E'}(\mathrm{t})-{\mathrm{c}_2}(\mathrm{t})\nonumber \\ \frac{{ - \mathrm{d}{\mathrm{V}_{\mathrm{E}'}}(\mathrm{t})}}{{\mathrm{dt}}}= &\, {} - {\mathrm{V}_{E'}}(\mathrm{t}) + {\mathrm{V}_{\mathrm{I}'}}(\mathrm{t})\nonumber \\ \frac{{ - \mathrm{d}{\mathrm{V}_{\mathrm{I}'}}(\mathrm{t})}}{{\mathrm{dt}}}= &\, {} - {\mathrm{V}_{\mathrm{I}'}}(\mathrm{t}) + {\mathrm{V}_{\mathrm{H}'}}(\mathrm{t})\nonumber \\ \frac{{ - \mathrm{d}{\mathrm{V}_{\mathrm{H}'}}(\mathrm{t})}}{{\mathrm{dt}}}= & \,{} - {\mathrm{V}_{\mathrm{H}'}}(\mathrm{t}) + {\mathrm{V}_{\mathrm{R}'}}(\mathrm{t})+{\mathrm{V}_{\mathrm{D}'}}(\mathrm{t})-1\nonumber \\ \frac{{ - \mathrm{d}{\mathrm{V}_{\mathrm{R}'}}(\mathrm{t})}}{{\mathrm{dt}}}= &\, {} 0\nonumber \\ \frac{{ - \mathrm{d}{\mathrm{V}_{\mathrm{D}'}}(\mathrm{t})}}{{\mathrm{dt}}}= &\, {} 0\nonumber \\ \frac{{ - \mathrm{d}{\mathrm{V}_{\mathrm{V}'}}(\mathrm{t})}}{{\mathrm{dt}}}= &\, {} 0 \end{aligned}$$

By solving above equations simultaneously, it is obtained ${\mathrm{V}_{\mathrm{E}'}} = -1,~{\mathrm{V}_{\mathrm{I}'}} =-\frac{({\mathrm{c}_1}(\mathrm{t})+{\mathrm{c}_2}(\mathrm{t}))}{2},~{\mathrm{V}_{\mathrm{H}'}} = -1,~{\mathrm{V}_{\mathrm{R}'}} = 0,~{\mathrm{V}_{\mathrm{D}'}} = 0,~{\mathrm{V}_{\mathrm{V}'}} = 0$ and have the following system:

9 $$\begin{aligned} \frac{{\mathrm{dS}'(\mathrm{t})}}{{\mathrm{dt}}}= &\, {} - (1 - \upalpha )\upsigma ({{{\overline{\mathrm{c}}}}_1})\mathrm{S}'(\mathrm{t})\mathrm{I}(\mathrm{t}) - \upalpha \mathrm{S}'(\mathrm{t}),\nonumber \\ \frac{{\mathrm{dM}'(\mathrm{t})}}{{\mathrm{dt}}}= & \,{} - \upsigma ({{{\overline{\mathrm{c}}}}_2})\mathrm{M}'(\mathrm{t})\mathrm{I}(\mathrm{t}),\nonumber \\ \frac{{\mathrm{dE}'(\mathrm{t})}}{{\mathrm{dt}}}= & \,{} (1 - \upalpha )\upsigma ({{{\overline{\mathrm{c}}}}_1})\mathrm{S}'(\mathrm{t})\mathrm{I}'(\mathrm{t}) + \upsigma ({{{\overline{\mathrm{c}}}}_2})\mathrm{M}'(\mathrm{t})\mathrm{I}'(\mathrm{t}) - \mathrm{E}'(\mathrm{t}),\nonumber \\ \frac{{\mathrm{dI}'(\mathrm{t})}}{{\mathrm{dt}}}= &\, {} \mathrm{E}'(\mathrm{t}) - \mathrm{I}'(\mathrm{t}),\nonumber \\ \frac{{\mathrm{dI}'(\mathrm{t})}}{{\mathrm{dt}}}= & \,{} \mathrm{I}'(\mathrm{t}) - \mathrm{H}'(\mathrm{t}),\nonumber \\ \frac{{\mathrm{dR}'(\mathrm{t})}}{{\mathrm{dt}}}= &\, {} \mathrm{H}'(\mathrm{t}),\nonumber \\ \frac{{\mathrm{dD}'(\mathrm{t})}}{{\mathrm{dt}}}= &\, {} \mathrm{H}'(\mathrm{t}),\nonumber \\ \frac{{\mathrm{dV}'(\mathrm{t})}}{{\mathrm{dt}}}= &\, {} \upalpha \mathrm{S}'(\mathrm{t}),\nonumber \\ \frac{{ - \mathrm{d}{\mathrm{V}_{\mathrm{S}'}}(\mathrm{t})}}{{\mathrm{dt}}}= & \,{} - ((1-\upalpha )\upsigma ({\mathrm{c}_1}(\mathrm{t})){\mathrm{I}'}(\mathrm{t})+\upalpha ){\mathrm{V}_{\mathrm{S}'}}(\mathrm{t}, {\mathrm{c}_1}, {{{\overline{\mathrm{c}}}}_1})-(1-\upalpha )\upsigma ({\mathrm{c}_1}(\mathrm{t})) \mathrm{I}'(\mathrm{t})-{\mathrm{c}_1}(\mathrm{t})\nonumber \\ \frac{{ - \mathrm{d}{\mathrm{V}_{\mathrm{M}'}}(\mathrm{t})}}{{\mathrm{dt}}}= & \,{} - \upsigma ({\mathrm{c}_2}(\mathrm{t}))\mathrm{I}'(\mathrm{t}){\mathrm{V}_{\mathrm{M}'}}(\mathrm{t},{\mathrm{c}_2},{{{\overline{\mathrm{c}}}}_2}) - \upsigma ({\mathrm{c}_2}(\mathrm{t})) \mathrm{I}'(\mathrm{t})-{\mathrm{c}_2}(\mathrm{t}) \end{aligned}$$

Now the purpose is finding ${\mathrm{c}_1}^*(\mathrm{t})$ and ${\mathrm{c}_2}^*(\mathrm{t})$, so it is used the maximum principle:

10 $$\begin{aligned} {\mathrm{c}_1}^*(\mathrm{t})= & {} \mathop {\arg \max }\limits _{\mathrm{c}_1}(\mathrm{t}) ((1-\upalpha )\upsigma ({\mathrm{c}_1}(\mathrm{t})){\mathrm{I}'}(\mathrm{t})+\upalpha ){\mathrm{V}_{\mathrm{S}'}}(\mathrm{t},{\mathrm{c}_1},{{{\overline{\mathrm{c}}}}_1} )+(1-\upalpha )\upsigma ({\mathrm{c}_1}(\mathrm{t}))\mathrm{I}'(\mathrm{t})+{\mathrm{c}_1}(\mathrm{t}), \end{aligned}$$

11 $$\begin{aligned} {\mathrm{c}_2}^*(\mathrm{t})= & {} \mathop {\arg \max }\limits _{\mathrm{c}_2}(\mathrm{t}) \upsigma ({\mathrm{c}_2}(\mathrm{t}))\mathrm{I}'(\mathrm{t}){\mathrm{V}_{\mathrm{M}'}}(\mathrm{t},{\mathrm{c}_2},{{{\overline{\mathrm{c}}}}_2}) +\upsigma ({\mathrm{c}_2}(\mathrm{t}))\mathrm{I}'(\mathrm{t})+{\mathrm{c}_2}(\mathrm{t}), \end{aligned}$$

12 $$\begin{aligned} {\mathrm{c}_1}^*(\mathrm{t})= & {} \sqrt{\frac{(1-\upalpha )\mathrm{I}'(\mathrm{t})({\mathrm{V}_{\mathrm{S}'}}(\mathrm{t},{\mathrm{c}_1},{{{\overline{\mathrm{c}}}}_1}) + 1)}{\mathrm{p}}} -1, \end{aligned}$$

13 $$\begin{aligned} {\mathrm{c}_2}^*(\mathrm{t})= & {} \sqrt{\frac{\mathrm{I}'(\mathrm{t})({\mathrm{V}_{\mathrm{M}'}}(\mathrm{t},{\mathrm{c}_2},{{{\overline{\mathrm{c}}}}_2}) + 1)}{\mathrm{p}}} -1. \end{aligned}$$

## Results and discussion

Infectious diseases are a threat to the health of the population. One of the easiest ways for individuals to reduce the risk of infection during an epidemic is to decrease their rate of contact with infected individuals. However, the value of these actions depends on how the epidemic progresses. Few cases analyses indicate how changes in behavior will change the epidemic wave. The policies included large-scale quarantine, strict controls on travel, and extensive monitoring of suspected cases, and as a rule, social distancing was applied by individuals and governments. In this study, two models have been studied. In the first model, the population was divided into seven possible states: Susceptible (S), Exposed (E), Infected (I), Hospitalized (H), Recovered (R), Death (D), and Vaccinated (V). The model is called, “SEIHRDV”. After getting infected, an individual enters the hospitalized compartment and at the end of this state either recovers or dies. The rates of changes in each state are, $\upbeta$ (S to E), $\upgamma$ (E to I), $\upnu$ (I to H), ${\updelta _1}$ (H to R), ${\updelta _2}$ (H to D) and $\upalpha$ (S to V). The average latent time, ${\upgamma ^{ - 1}}$ is considered 2 days, so the rate of transmission, between E to I was considered $\upgamma = 0.5$. The hospitalized time is considered 14 days and therefore ${\upnu ^{ - 1}}$ is set to 0.07. The infectious rate, the recovered rate and the mortality rate are defined as the ratio of the number of daily diagnosed COVID-19 patients at time $\mathrm{t}$ to the number of accumulated COVID-19 patients at time $\mathrm{t}$, the ratio of the number of daily recovered COVID-19 patients at time $\mathrm{t}$ to the number of accumulated COVID-19 patients at time $\mathrm{t}$, and the ratio of the number of daily died COVID-19 patients at time $\mathrm{t}$ to the number of accumulated COVID-19 patients at time $\mathrm{t}$, respectively. These parameters were estimated using WHO data for each country. In order to reduce and control the spread of COVID-19, social distancing is a non-pharmaceutical strategy that was considered in the model and the system (2) modified to the system (3). This system solved numerically by using the estimated parameters in Table 2. Social distancing is an attitude of behavior and changing the behaviors can decrease contact rates and that makes to reduce the transmission of infectious and control the diseases. The number of confirmed death per day published by several media can affect the behavior of individuals in social distancing. Therefore, here we approximate the social distancing effect as the ratio of the number of daily died at time $\mathrm{t}$ to the number of accumulated patients at time $\mathrm{t}$.

**Table 2:** Parameters estimated from the observed data in Germany (DE), Italy (IT), Belguim (BE), Egypt (Eg), Nigeria (NG) and Japan (JP) inferred from the World Health Organization.

Parameters	Value-DE	Value-IT	Value-BE	Value-EG	Value-NG	Value-JP
$\mathrm{N}\, (\hbox {million})$	80	60	11.5	102	195	126.5
${\upgamma ^{ - 1}}$	2	2	2	2	2	2
${\upnu ^{ - 1}}$	14	14	14	14	14	14
${\mathrm{E}_0}$	2	2	2	2	2	2
${\mathrm{I}_0}$	1	2	1	1	1	1
${\mathrm{H}_0}$	0	0	0	0	0	0
${\mathrm{R}_0}$	0	0	0	0	0	0
${\mathrm{D}_0}$	0	0	0	0	0	0
${\mathrm{V}_0}$	0	0	0	0	0	0

**Figure 3:**
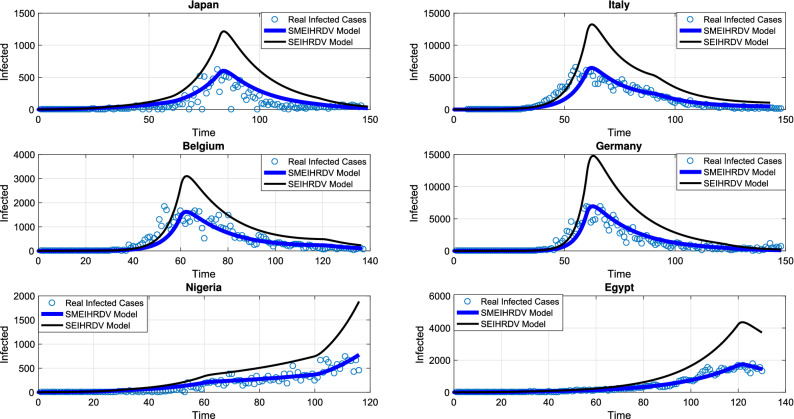
Fitting the models with real data. The number of infected individuals predicted by the “SEIHRDV” and the “SMEIHRDV” models for Japan (from 22th January to 23th June 2020), Italy (from 31th January to 23th June 2020), Belgium (from 4th February to 23th June 2020), Germany (from 27th January to 23th June 2020), Nigeria (from 28th February to 23th June 2020) and Egypt (from 14th February to 23th June 2020). The blue dots denote the reported infected cases and the black (or Less width) and blue lines present the results of the “SEIHRDV” and the “SMEIHRDV” models, respectively. The deSolve package version 1.28^27^ in R software version 4.0.3^28^ was used to generate the figure (http://desolve.r-forge.rproject.org/).

In the second model, we assume that some part of population have lower susceptibility to infection. In general, factors such as age, smoking, obesity and genetic factors can affect disease susceptibility^26^. Therefore, we added a new compartment called semi-susceptible denoted by M, and assume that their infection rate to COVID-19 is half the infection rate of susceptible people to it. The new model is SMEIHRDV. In this new model, for each country, ${\mathrm{M}_0}=\frac{15}{100} \mathrm{N}$ and the rates of changes in states are, ${\upbeta _1}$ (S to E), ${\upbeta _2 =\frac{1}{2} \upbeta _1}$ (M to E) and the remaining parameters are the same as the SEIHRDV model. Social distancing was considered in this model and the system (4) was solved numerically, by using the estimated parameters in Table 2. The confirmed and predicted infected cases of the SMEIHRDV and SEIHRDV models is shown in Fig. 3, for Japan (from 22th January to 23th June 2020), Italy (from 31th January to 23th June 2020), Belgium (from 4th February to 23th June 2020), Germany (from 27th January to 23th June 2020), Nigeria (from 28th February to 23th June 2020) and Egypt (from 14th February to 23th June 2020). The blue dots denote the reported infected cases and the black (or less width) and blue lines represent the model simulation results, respectively. The comparison of the results of two models shows that the SMEIHRDV model provides better estimation with the reported WHO data. Therefore, the semi-susceptible compartment improves the result of the model in the prediction of infected individuals.

**Figure 4:**
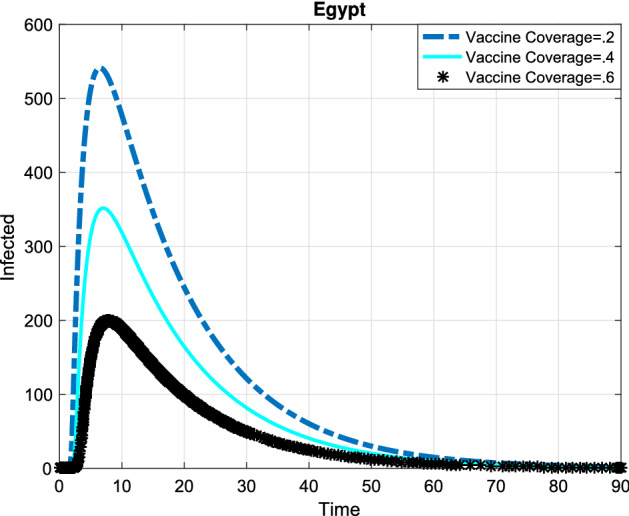
Simulation, the forecast of the epidemic process. The forecast of the epidemic growing tendency for infected cases through the SMEIHRDV model in Egypt. The results show that by increasing the vaccine coverage, the epidemic peak for infected cases decreases. The number of infected cases at the epidemic peak is approximately 540 in Egypt and it has reduced to 200 by the increasing vaccine coverage from 0.2 to 0.6. $\hbox {N}=102,000,000.$

**Figure 5:**
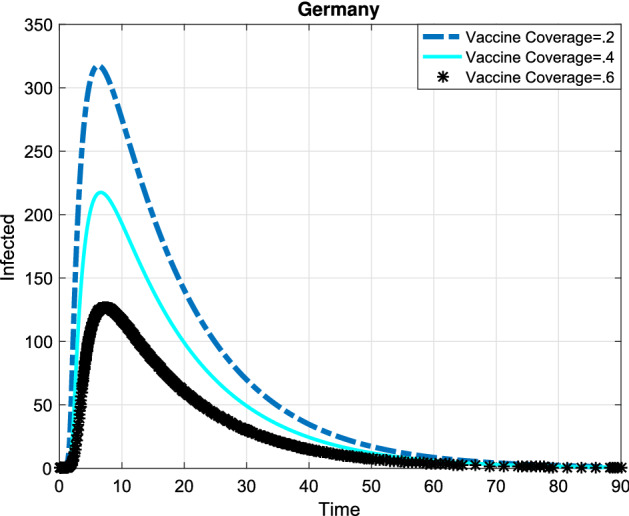
Simulation, the forecast of the epidemic process. Infected cases are predicted by the SMEIHRDV model in Germany. The results show that the number of infected cases at the epidemic peak is approximately 320 in Germany and it has reduced to 120 by the increasing vaccine coverage from 0.2 to 0.6. $\hbox {N}=80,000,000.$

**Figure 6:**
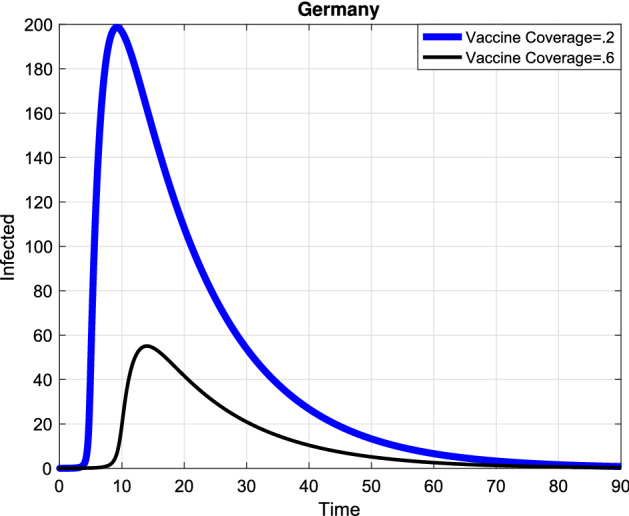
Simulation, the forecast of the epidemic process. The forecast of the epidemic growing tendency for infected cases through the SMEIHRDV model with the best investment strategy in social distancing. The results show that the best investment strategy in social distancing reduces the epidemic peak for infected cases and also by increasing the vaccine coverage, the epidemic peak for infected cases decreases. The number of infected cases at the epidemic peak is approximately 200 in Germany and it has reduced to 55 by the increasing vaccine coverage from 0.2 to 0.6. $\hbox {N}=80,000,000.$

In the following, the game theory of social distancing was presented and in order to obtain the best investment strategy in social distancing, we used the dimensionless version of the dynamic the system (5) and a differential game for a susceptible individual choosing his or her best investment strategy in social distancing relative to the aggregate investment strategy of susceptible population as whole and a differential game for a semi-susceptible individual choosing his or her best investment strategy in social distancing relative to the aggregate investment strategy of semi-susceptible population as whole. Since the exact time spent and the precise payoff are not predicted, so we used the Markov process. The system (6) that is a dimensionless version of the SMEIHRDV model, was used to determine the transition-rate matrix. This model was simulated to investigate the effect of vaccination and the investment in social distancing to drop the epidemic peak. The aggregate investment strategy of susceptible individuals denoted by ${{{\overline{\mathrm{c}}}}_1}(\mathrm{t})$ and the aggregate investment strategy of semi-susceptible denoted by ${{{\overline{\mathrm{c}}}}_2}(\mathrm{t})$. Here, it was used the maximum principle to find the best strategy of the investment in social distancing for a susceptible individual (${\mathrm{c}_1}^*$) and for a semi-susceptible individual (${\mathrm{c}_2}^*$), and they were obtained in (12) and (13). Finally, the SMEIHRDV model was simulated for when the vaccine was released for Coronavirus. We investigate the effect of the behavior of individuals in vaccination in the epidemic process. The different vaccine coverage were examined in the epidemic process for the infected cases in Egypt (see Fig. 4) and Germany (see Fig. 5). The number of total infected individuals ( at the epidemic peak) in Egypt decreased from 540 to 200 as the vaccination coverage changes from 0.2 to 0.6 (see Fig. 4). Also, in Germany, the number of infected cases at the epidemic peak is approximately 320 and it has reduced to 120 by the increasing vaccine coverage from 0.2 to 0.6 (see Fig. 5). In Fig. 6, the effect of the best investment strategy in social distancing was studied in COVID-19 progress in Germany; the forecast of the epidemic growing tendency for infected cases through the SMEIHRDV model with the best investment strategy in social distancing. The results show that the best investment strategy in social distancing reduces the epidemic peak for infected cases and also by increasing the vaccine coverage, the epidemic peak for infected cases in Germany decreases. The number of infected cases at the epidemic peak is approximately 200 and it has reduced to 55 by the increasing vaccine coverage from 0.2 to 0.6.
